# Investigation of the Hydration Behavior of Different Sugars by Time Domain-NMR

**DOI:** 10.3390/foods11081148

**Published:** 2022-04-15

**Authors:** Ozan Tas, Ulku Ertugrul, Leonid Grunin, Mecit Halil Oztop

**Affiliations:** 1 Department of Food Engineering, Middle East Technical University, Ankara 06800, Turkey; ozantas@metu.edu.tr (O.T.); uelkue.ertugrul@partner.kit.edu (U.E.); 2 Department of Food Chemistry and Phytochemistry, Institute of Applied Biosciences, Karlsruhe Institute of Technology (KIT), 76131 Karlsruhe, Germany; 3 Food Process Engineering, Institute of Process Engineering in Life Sciences, Karlsruhe Institute of Technology (KIT), 76131 Karlsruhe, Germany; 4 Resonance Systems GmbH, 73230 Kirchheim unter Teck, Germany; mobilenmr@hotmail.com

**Keywords:** TD-NMR, allulose, hydration, self-diffusion coefficient, magic sandwich echo

## Abstract

The hydration behavior of sugars varies from each other and examining the underlying mechanism is challenging. In this study, the hydration behavior of glucose, fructose, allulose (aka rare sugar), and sucrose have been explored using different Time Domain Nuclear Magnetic Resonance (TD-NMR) approaches (relaxation times, self-diffusion, and Magic Sandwich Echo (MSE)). For that purpose, the effects of different sugar concentrations (2.5%, 5%, 10%, 15%, 20%, 30%, and 40%) (*w*/*v*) and hydration at different times for 1 day were investigated by T_2_ relaxation times and self-diffusion coefficients. Crystallinity values of the solid and hydrated sugars were also determined with MSE. Change in T_2_ relaxation times with concentration showed that the fastest binding with water (parallel with the shortest T_2_ values) was observed for sucrose for all concentrations followed by glucose, fructose, and allulose. Furthermore, dependency of T_2_ relaxation times with hydration time showed that sucrose was the fastest in binding with water followed by glucose, fructose, and allulose. The study showed that allulose, one of the most famous rare sugars that is known to be a natural low-calorie sugar alternative, had the lowest interaction with water than the other sugars. TD-NMR was suggested as a practical, quick, and accurate technique to determine the hydration behavior of sugars.

## 1. Introduction

The word “sugar” recalls tiny white crystals that are recognized as sweet molecules. Sugars are not only used as sweeteners but also have crucial functions in food products as they provide texture, bulkiness, color, and act as preservatives [1].

Sugars are known as humectants which means they highly interact with water. This interaction has a critical impact on the functional properties such as solubility, water activity, glass transition temperature, osmotic pressure, and hydration which are highly important in most food applications [2]. Being solid particles and polar molecules due to the presence of -OH groups in the structures allows sugar molecules to interact with water by forming hydrogen bonds [3]. This interaction between sugars and water is called sugar hydration. Sugar hydration has significant importance in many food applications, especially for shelf-life determination and the preservation of sugar-rich food products [4,5]. Besides being so important for many applications, it is very difficult to understand the mechanism underlying the sugar–water interactions. For that reason, many studies continue to examine the science behind sugar hydration. In one of the studies, it was pointed out that sugar hydration depends on the type of branching, the linkage between monomers, and the anomeric configuration in the structure of the sugars [6]. Furthermore, it was stated that sugar hydration is directly affected by the number of equatorial -OH groups of the sugar molecules [7]. In another study that was conducted on fructose, glucose, and mannose, it was observed that small differences in the stereochemistry of sugars cause different hydration behaviors of these molecules. According to the findings, the average hydrogen bond length between the sugars and water molecules is correlated with hydration of the sugars [8].

Allulose is one of the rare sugars that has received attention due to its many benefits in recent years. It has high solubility, has a desired sweet taste, and is lower in calories compared to the other monosaccharides [9,10]. There is still not much information regarding the interaction of allulose with water. Therefore, the hydration behavior of this sugar is worth investigating to determine its desired use in food formulations.

Time Domain Nuclear Magnetic Resonance (TD-NMR) relaxometry is defined as an accurate, reliable, quick, harmless, and practical tool that has been employed to gain structural and dynamic information on a molecular level in food components. In addition, it gives ideas about the water uptake, water content, and distribution in a sample [11,12]. There are many food-related applications in which TD-NMR relaxometry is used. Lipids, seeds, meat products, fruit and vegetables, beverages, and sugar-containing systems are just some of the examples [13,14,15,16].

With the help of TD-NMR relaxometry, the water and solute interactions can be determined by looking at the spin–spin (T_2_) and spin–lattice (T_1_) relaxation times. It is a known fact that these times change depending on the status of water in the system [17]. The decrease in these times indicates a decrease in the amount of free water in a system [18]. When the comparison between those two times was made, it was seen that T_1_ times might be 5 to 10 times longer than T_2_ times. In that regard, the rate of decrease in T_2_ relaxation times can be used to evaluate and compare the hydration behavior of different types of sugars, since T_2_ times give quicker results.

In addition to that, some alternative NMR experiments such as measuring self-diffusion coefficients of water in sugar solutions could also give valuable information about sugar-water interaction. The self-diffusion coefficient is one of the transport-related properties of water and it has a crucial role in the physical and chemical explanation of water’s interaction with different molecules [19]. There are many studies that used the pulsed-field gradient NMR (PFG-NMR) to study the self-diffusion of water in porous food systems. Some of the examples can be given as cheeses, bread matrices, and different types of food gels [20].

Sugars that are used in foods are mostly found in crystalline forms and the degree of crystallinity is also a parameter that can affect how water will interact with the sugar. The crystallinity of powders is mostly obtained through X-ray powder diffraction experiments [21]. Free Induction Decay (FID) sequence, which is known as the basic NMR signal obtained by a 90° radiofrequency (RF) pulse, has been used to measure the crystal content of some sugars such as sucrose, sorbitol, and powder sugar [22,23,24,25]. However, when using this basic sequence, there is a risk of a loss in the signal coming from the solid part because of the dead time in the sequence. Deadtime is defined as the time that is lost in the RF probe till the first point of the signal is obtained. To eliminate the problem of dead time, alternative sequences such as Solid Echo (SE) and Magic Sandwich Echo (MSE) were developed [26]. Although the SE sequence is more reliable than the FID sequence, it is not capable of fully refocusing on the multiple dipolar interactions. In that regard, the MSE sequence which is the modified form of SE sequence can be used to determine the crystallinity of a substance since it enables nearly the full refocus on the multiple dipolar interactions with correct parameters [27,28].

The present study focuses on showing and comparing the hydration behavior of glucose, fructose, allulose, and sucrose sugars by several TD-NMR approaches. For this, T_2_ values, self-diffusion coefficients, and the crystallinity of sugars by Magic Sandwich Echo (MSE) experiments were evaluated. This study hypothesizes that TD-NMR relaxometry can be a reliable and practical tool to explain the hydration behavior of the sugars to a large extent.

## 2. Materials and Methods

### 2.1. Materials

Glucose, fructose, and sucrose sugars were purchased from Sigma-Aldrich Chemical Co., Ltd. (St. Louis, MO, USA). Allulose was purchased from Lang’s Chocolates (Williamsport, PA, USA). The purity of all purchased sugars was stated as ≥99% (GC).

### 2.2. Methods

#### 2.2.1. Determination of Hydration Behavior by TD-NMR Relaxometry

Experiments were performed at 298 K by using two benchtops TD-NMR systems (Spin Track, Resonance Systems GmbH, Kirchheim unter Teck, Germany) that are 0.48 Tesla, operating at a 1 H frequency of 20.34 MHz and 0.32 Tesla, operating at a 1 H frequency of 13.52 MHz.

##### Spin–Spin Relaxation Time (T_2_)

First, to eliminate the effect of particle size, all sugars were ground with a blender and sieved to obtain the same particle size for all sugars. T_2_ measurements were conducted in two different sets:

First Set: Sugar solutions at different concentrations were prepared and (2.5%, 5%, 10%, 15%, 20%, 30% and 40%) (*w*/*v*) T_2_ relaxation times (s) were measured. Then, relaxation rates (1/T_2_) were calculated and shown at different concentrations for all sugar types.

Second Set: The effect of time on sugar hydration was evaluated by mixing 3 g of sugars in 1 g of distilled water. Then, T_2_ relaxation times (ms) were measured at different times for 1 day.

For both sets, the data of T_2_ relaxation times measurements were conducted using a CPMG (Carr–Purcell–Meiboom–Gill) pulse sequence with an echo time of 1000 ms, 4 s repetition time, 16 scans, and the number of echoes changed between 2000–4000. The analysis was conducted using MATLAB (The MathWorks Inc., MA, USA) considering a mono-exponential relaxation behavior. A characteristic decay curve is provided in the Supplementary Materials.

(1) $$M_{xy}\left(t\right)=M_{0}\;\left(e^{-{\frac{t}{T}}_{2}}\right)$$

##### Self-Diffusion Coefficient

The diffusion coefficients of the water molecules inside sugar solutions, prepared at different concentrations mentioned above, were analyzed. For that, a special module in RELAX 8 (Resonance Systems, Kirchheim unter Teck, Germany) was used. The relaxation period was set as 10^4^ ms and the number of scans was set as 64 and the diffusion time was set as 100 ms. Classical Stimulated Echo sequence was applied with a variation of the gradient strength from 0 to 2 Tesla per meter. Gradient pulse duration was set to 2 ms. Stimulated echo attenuation was plotted versus squared gradient values and fitted with a single exponential function. Calibration of the gradient strength was completed with distilled water that had a known self-diffusion coefficient [29,30].

##### Use of Magic Sandwich Echo (MSE) Sequence for Crystallinity Measurements

The NMR line second moment M_2_, that is proportional to crystallinity [25] values of the sugars in solid and hydrated (sugars were mixed with water at the ratio of 3/1 of their weight waited for 1 day) forms, was analyzed by Free Induction Decay (FID) recorded by MSE sequences. For this, the relaxation period was set as 10^4^ ms, and the number of scans was set as 16. The data of MSE were analyzed by using the special module in RELAX 8 (Resonance Systems, Kirchheim unter Teck, Germany). The analysis was based on the averaging of the three regions of the FID: short (S) (0–2 us), medium (M) (10–16 us), and long (L) (60–80 us). It was supposed that the L represented information of residual moisture content. M–L and S–M represent amorphous and crystalline contributions, respectively. Calibration of the method was completed with Gaussian and Abrahamian numerical models of FID and they both showed equal and linear dependence of the value (S–M)/(S–L) on the second moment (M_2_) of calibration models in the range of 0 to 30 × 10^−8^ Tesla^2^ and the M_2_ values were calculated as (S–M)/(S–L).

In addition, the relative total crystallinity (%) of the sugars in solid state was also measured by using X-ray Diffraction (XRD) (Rigaku Ultima-IV X-Ray Diffractometer, Woodlands, TX, USA) at METU Central Laboratory. The sampling width, scan axis, range, and speed were set as 0.02°, 20, 5–50°, and 1°/min, respectively. X-ray diffractograms are provided in the Supplementary Materials.

#### 2.2.2. Statistical Analysis

All experiments were conducted for three replicates and statistical analysis was performed by MINITAB (Version 19, Minitab Inc., Coventry, UK). Analysis of variances (ANOVA) was executed to observe the effect of different variables on the results by using a general linear model regression. Before the comparisons, assumptions of ANOVA such as normality of the residuals, constant variance, independence, and multicollinearity were examined, and transformations were completed when necessary. Tukey’s comparison test at a 95% confidence interval was performed to evaluate the significance when necessary.

## 3. Results and Discussion

### 3.1. Spin-Spin Relaxation Times (T_2_)

#### 3.1.1. Effect of Sugar Concentration on the Relaxivity of Solutions

Spin–spin relaxation times denoted as T_2_ give information about the state of water (bound/free, mobile/immobile) and the interaction of water with surrounding molecules [31,32]. T_2_ times are usually measured by a CPMG pulse sequence and are known to be a quick and reliable indicator to assess water solute interactions [18,33]. T_2_ relaxation times are expressed in terms of relaxation rates; 1/T_2_ when explaining the changes with respect to concentrations. Relaxivity is defined as the change in the relaxation rates of a solution as a function of concentration and it has been shown to provide meaningful information regarding the hydration of a substance [34,35]. In addition, relaxivity is directly proportional to the hydration number since an increase in hydration number causes an increase in relaxivity values [36].

**Figure 1:**
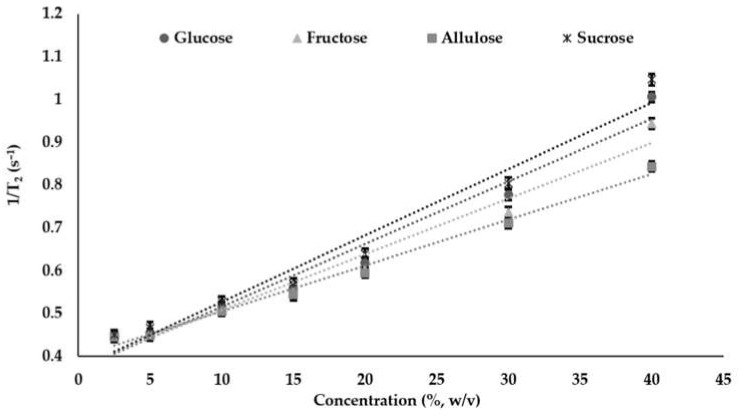
Relaxation rates (1/T_2_) (s^−1^) of sugars at different concentrations (%) (Data for 1/T_2_ are expressed as mean ± SE (*n* = 3)).

In this study, the relation between relaxation rate and concentration was fitted to a linear model (Figure 1) and the equations of the models are shown in Table 1. Relaxivity values were obtained as the slopes of the relaxation rates (1/T_2_) and they showed significant differences with the values as 0.0146, 0.0130, 0.0107, and 0.0155 for glucose, fructose, allulose, and sucrose, respectively (100 mL/g-s). According to the results, sucrose had the highest relaxivity value followed by glucose, fructose, and allulose, respectively (*p* < 0.05). Besides, this trend was obtained for all concentrations from 2.5% to 40% (*p* < 0.05). In addition, for all sugar types, as the concentration increased, relaxivity values increased due to more sugar–water interaction in which the free water in the system decreased as expected. The outcome of these results can be interpreted as the sugars showing the same sensitivity for the change in concentrations. Apart from that, sucrose showed the highest interaction with water, namely, it was more hydrated than the other sugars. In some of the studies, hydration of sucrose was evaluated by considering the molecular dynamics such as the hydration number. It was shown that sucrose had a relatively large hydrodynamic size due to hydroxylic and acetalic oxygens in the structure which led to a high tendency to make a hydrogen bond with water [37,38]. Furthermore, in the study where glucose and fructose sugars were evaluated and compared in the case of hydration behavior, it was shown that glucose had more tendency to hydrate than fructose, which was explained by the molecular ring size and the fractions of axial and equatorial -OH groups in the structures being different [39]. Among the sugar types, the least hydrated sugar was allulose based on the relaxivity values obtained (*p* < 0.05). In one study, the hydration behavior of allulose, also known as D-Psicose, was determined and compared with other sugars by checking dielectric relaxation [40]. The results were determined by using average hydration number and water activity values. In the conclusion of the study, both fructose and D-Psicose were shown to have interacted similarly with water. However, our study results suggested a different behavior for allulose having less affinity with water, which was also observed in other studies where allulose was used as a sugar substitute in confectionery gels [14,41].

Mutarotation also has a significant impact on the hydration behavior of sugars since in solutions, reducing sugars’ exchange between their anomers and may result in a different hydration behavior [42]. In this study, all the sugars except sucrose (as being non-reducing) can mutarotate in the solution due to the presence of the reducing end. In the native forms, it was shown that D-glucose was found in the α-pyranose crystal form [43] while D-fructose and D-allulose had only one crystal form that was β-pyranose [26,44]. When these sugars are hydrated, they interchange between their tautomers and the occurrence of different tautomeric ratios significantly affects the hydration behavior of sugars [45]. In aqueous solutions, D-glucose exists in α-pyranose (31.1–37.4%) and β-pyranose (64–67.9%) forms, while D-fructose is in α-pyranose (≈4%), β-pyranose (68.4–76.0%), and β-furanose (28.0–31.6%) forms at ambient temperature. Besides, for D-allulose at 27 °C, four different forms could be found, which are α-furanose (39%), β-furanose (15%), α-pyranose (22%), and β-pyranose (24%) [26]. Studies confirmed that the β anomers showed higher numbers of water oxygens in the first hydration shell compared to the α anomers. This was explained by the improved water interactions of β anomers having the anomeric oxygen in equatorial form.

In this study, solutions of glucose, fructose, and allulose at ambient temperature were investigated and it was observed that allulose, having more heterogeneous tautomers and less β-pyranose forms, had reduced binding ability towards water molecules compared to glucose and fructose. Besides, although two ketoses (fructose and allulose) are epimers of each other, the number of hydroxyl groups in the equatorial position are different in their β pyranose forms; two (C3 and C4) for fructose and one (C4) for allulose [46]. This fact may have also caused allulose to have the least interaction with water compared to other sugars.

#### 3.1.2. Change in T_2_ Relaxation Times with Time

**Figure 2:**
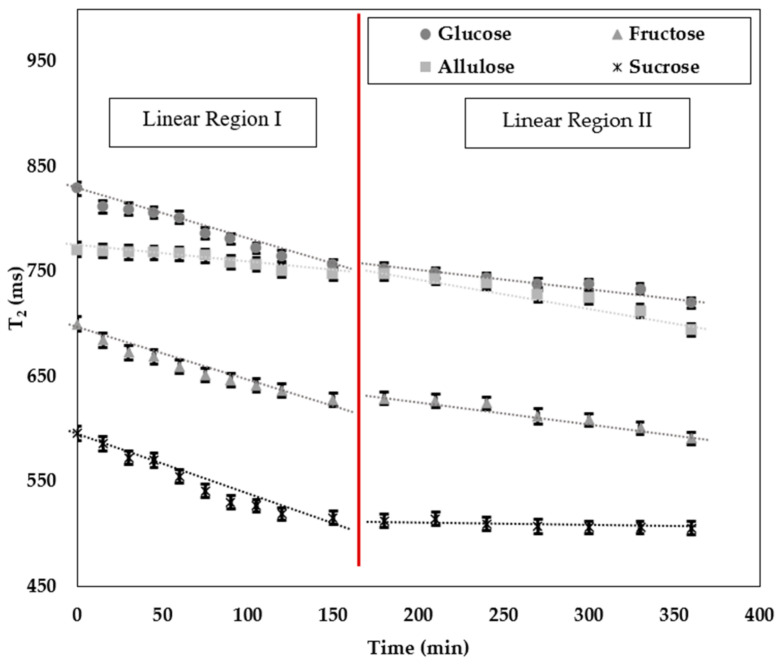
T_2_ (ms) relaxation times of sugars obtained at different times. Data for T_2_ times are expressed as mean ± SE (*n* = 3).

Hydration is the process in which the water penetrates and diffuses within the solid molecules. Furthermore, the amount of water absorbed increases as the process time increases [47]. Thus, time is one of the most important variables for the hydration of the molecules. In this study, the hydration rate of the sugars was observed by TD-NMR relaxometry for 1 day (Figure 2). Preliminary experiments showed that after 400 min, there were slight changes in the T_2_ relaxation times, so hydration was nearly completed for all sugars by that time. Figure 2 displays two different regions (region I and region II) for which linear model fittings were applied.

To compare the hydration rate of the sugars, the slopes of the curves were taken into consideration (Table 2). According to the ANOVA results, the highest hydration rate belonged to sucrose followed by glucose, fructose, and allulose for the region I (*p* < 0.05). Furthermore, sucrose showed the lowest hydration rate in the region II (*p* < 0.05). Thus, it can be stated that sucrose was the fastest in water binding since it absorbed the water in 150 min, and there was a little amount of water absorption after that time, as seen in region II. Apart from that, allulose was the only sugar that showed a higher hydration rate in the region II, indicating that hydration was still ongoing after 150 min. In most of the studies, the structural changes in the sugar molecules, especially the position and the distance between the -OH groups within the structures, had huge impacts on reaction rate in hydration [48,49]. In one study, the effect of intramolecular hydrogen bonds on sucrose hydration was studied and it was found that sucrose showed rare intramolecular hydrogen bond formation, which indicates more mobility in the aqueous solution [37]. Considering the disaccharide nature of sucrose, water molecules might have been more quickly bounded. Furthermore, glucose was also shown to have higher hydration behavior due to its structural conformation where -OH groups are positioned to have more water interactions [50]. That is why sucrose and glucose could have been hydrated more quickly than fructose and allulose. The accessibility to -OH groups in ketoses could also be harder than the aldoses [51]. Thus, for our ketose sugars (fructose and allulose), the time for complete hydration was longer than the aldoses.

### 3.2. Self-Diffusion of Water in Different Sugar Solutions

**Figure 3:**
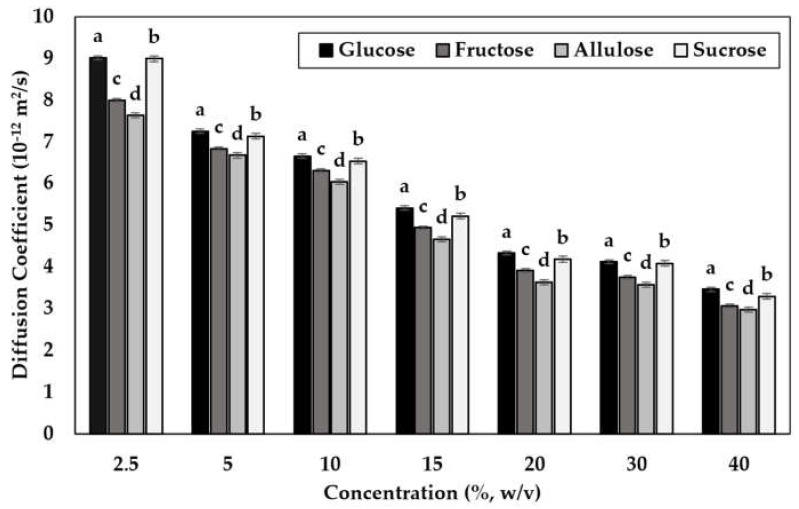
Self-diffusion coefficient of water in sugar solutions at different concentrations (%) obtained from Stimulated Echo Sequence (*w/v*). For each concentration value, lower case superscript letters (a–d) denote significant difference at 5% (*p* < 0.05). Values are expressed as mean ± SE (*n* = 3).

NMR can be used to calculate the self-diffusion coefficients of water in the range between 10^−6^ and 10^−14^ m^2^ s^−1^ in which the higher coefficient values indicate viscous liquids [52]. In this study, the self-diffusion coefficient of water in the sugar solutions was found to be significantly different (*p* < 0.05) at different concentrations and for different sugars (Figure 3).

By looking at Figure 3, the highest self-diffusion coefficient values were found for glucose followed by sucrose, fructose, and allulose at ambient temperature (298 K) (*p* < 0.05). In addition, this trend was the same for all concentration values indicating that increasing concentration did not affect the change in the self-diffusion of water in the sugar solutions. Determining the self-diffusion coefficients based on different sugar solutions was studied to a large extent in the literature, however, they were mainly for sucrose and glucose [53]. The findings showed that compared to solutions of sucrose, solutions including glucose had a slightly higher self-diffusion coefficient. The reason behind this was explained by the fact that the molecular weight of those two sugars is different. Indeed, sucrose is a disaccharide and may increase the viscosity of a solution, so can decrease the mobility of water in the solution more compared to glucose which is a monosaccharide [54]. Nevertheless, results obtained by the NMR showed that the self-diffusion coefficients of water in sucrose and glucose were so close to each other. Apart from that, there were not many studies in the literature regarding the self-diffusion coefficient of water in allulose. Our findings concluded that allulose solutions had the lowest self-diffusion coefficient value although allulose solutions had longer T_2_ values at all concentrations compared to other sugars. This finding confirmed that allulose is likely to form a network in the solution and create a tortuous path for the water molecules to move and restrict the diffusion. Moreover, as also stated in Section 3.1, allulose had various types of tautomer forms in their solutions at ambient temperature which might have resulted in a lower self-diffusion coefficient. This can be explained by the fact that in the equilibrium state of the solution, different tautomers might have competed during diffusion; thus, the movement of the water inside the allulose may have been retarded.

### 3.3. Crystallinity of Sugars by Magic Sandwich Echo (MSE) Sequence and X-ray Diffraction (XRD)

In this study, the Magic Sandwich Echo (MSE) sequence was used to obtain the crystallinity values of the sugars before and after hydration. Sugars were mixed with water at the ratio of 3/1 of their weight and equilibrated for 1 day, so surface water was left to form on the sugar crystals. A parameter named as second moment (M_2_) is correlated with crystallinity values and it was calculated as explained in Section 2, and the results are provided in Table 3.

When Table 3 was examined, after water was added to sugars and waited for 1 day, crystallinity values decreased for all sugars as sugar–water interaction occurred (*p* < 0.05). Non-hydrated (solid) sucrose initially had the highest crystallinity than the other sugars (*p* < 0.05). This was consistent with the fact that sucrose is more crystal in solid form than the other sugars such as glucose and fructose [55]. The crystallinity of glucose was also found to be higher than fructose in the solid form (*p* < 0.05). To confirm the results obtained from MSE experiments, X-ray Diffraction (XRD) analysis was also conducted on the solid forms of the sugars. The relative total crystallinity (%) of sugars was calculated and provided in Table 3. Moreover, the XRD plots are also given in the Supplementary Materials as Figure S1. According to the results, the highest crystallinity was observed again in sucrose, followed by allulose, glucose, and fructose, respectively (*p* < 0.05). When the results for solid forms of sugars obtained by both MSE sequence and XRD analysis were statistically compared, the correlation was found to be significant with a value of +0.828 (*p* < 0.05). Therefore, these results suggested that these experiments complied well with each other.

When the hydrated results were analyzed, it was seen that the highest crystallinity belonged to allulose, followed by fructose, glucose, and sucrose, respectively (*p* < 0.05). Allulose having the highest crystallinity following water addition was an indication that it had the lowest hydration since it did not lose its crystal form as much as the other sugars. In addition, sucrose had the lowest crystallinity value than the other sugars in the hydrated form, hence had the highest sugar–water interaction (*p* < 0.05). In one of the studies, glucose, lactose, and sucrose sugars were processed in freeze and spray dryers to obtain more hygroscopic and amorphous sugars [25]. According to the crystallinity results which were measured by MSE sequences, sucrose had the lowest crystallinity values than glucose and lactose in the amorphous state, which confirmed our findings that, after the water addition, the lowest crystallinity belonged to sucrose since it bounded more water than the other sugars.

## 4. Conclusions

Although sugar hydration has significant importance in many food applications, it is very difficult to understand the mechanism underlying the hydration process. TD-NMR relaxometry offers quick, accurate, and efficient information regarding sugar–water interactions, namely sugar hydration. In this study, four different sugars (glucose, fructose, allulose, and sucrose) were hydrated and the results were compared with several approaches of TD-NMR relaxometry. Different concentrations (2.5%, 5%, 10%, 15%, 20%, 30%, and 40%) (*w*/*v*), and time dependency of sugars concerning T_2_ relaxation times, self-diffusion coefficients of water, and crystallinity values by MSE experiments were obtained. The general findings from all experiments showed that sucrose showed the highest interaction with water followed by glucose, fructose, and allulose. This is the first study that has explored the hydration behavior of allulose, the famous rare sugar, with TD-NMR relaxometry. Results of the study can provide important guidance for the studies that use allulose in different food formulations. Further investigation could be made to reveal the effects of parameters such as temperature and the composition of the food formulations, to observe the hydration behavior of these sugars in processed food products.

## Figures and Tables

**Table 1:** Model fitting for relaxation rates, 1/T_2_ (s^−1^) with respect to sugar concentration in the solution.

Sugar Type	1/T_2_ (s^−1^)	R^2^
Glucose	1/T_2_ = 0.0146x + 0.370 ^b^	0.967
Fructose	1/T_2_ = 0.0130x + 0.379 ^c^	0.969
Allulose	1/T_2_ = 0.0107x + 0.398 ^d^	0.989
Sucrose	1/T_2_ = 0.0155x + 0.373 ^a^	0.968

Lower case superscript letters (a–d) denote significant difference at 5% (*p* < 0.05). Values are expressed as mean ± SE (*n* = 3).

**Table 2:** Model fitting of T_2_ (ms) with respect to time for regions I and II.

	Region I	R^2^	Region II	R^2^
Glucose	T_2_ = −0.478x + 824 ^b^	0.976	T_2_ = −0.163x + 783.341 ^c^	0.929
Fructose	T_2_ = −0.461x + 690 ^c^	0.959	T_2_ = −0.217x + 672.072 ^b^	0.963
Allulose	T_2_ = −0.162x + 773 ^d^	0.916	T_2_ = −0.280x + 802.703 ^a^	0.934
Sucrose	T_2_ = −0.587x + 591 ^a^	0.995	T_2_ = −0.048x + 521.911 ^d^	0.811

For each column, lower case superscript letters (a–d) denote significant difference at 5% (*p* < 0.05). Values are expressed as mean ± SE (*n* = 3).

**Table 3:** Second Moment (M_2_) results by MSE sequence and Relative Total Crystallinity (%) of sugars measured by XRD.

Sugar Type	M_2_ (10^−8^ Tesla^2^) (Solid)	M_2_ (10^−8^ Tesla^2^) (Hydrated)	Relative Total Crystallinity (%)
Glucose	16.11 ± 0.04 ^c^	13.64 ± 0.03 ^c^	76.72 ± 0.14 ^c^
Fructose	15.18 ± 0.03 ^d^	14.24 ± 0.02 ^b^	75.73 ± 0.16 ^d^
Allulose	16.39 ± 0.04 ^b^	14.41 ± 0.04 ^a^	79.71 ± 0.11 ^b^
Sucrose	16.47 ± 0.06 ^a^	13.15 ± 0.02 ^d^	81.30 ± 0.21 ^a^

For each column, lower case superscript letters (a–d) denote significant difference at 5% (*p* < 0.05). Values are expressed as mean ± SE (*n* = 3).

## Data Availability

The data presented in this study can be requested from the web page of the relevant project: https://suchaquality.com/.

## Supplementary Materials

The following supporting information can be downloaded at: https://www.mdpi.com/article/10.3390/foods11081148/s1, Figure S1: X-ray Diffaction (XRD) spectrum of sugars in solid form.
