# Supplementary figures and images for: Investigation of the Hydration Behavior of Different Sugars by Time Domain-NMR
Source: Foods. 2022 Apr 15;11(8):1148. doi: 10.3390/foods11081148 (PMC9031088; doi:10.3390/foods11081148)

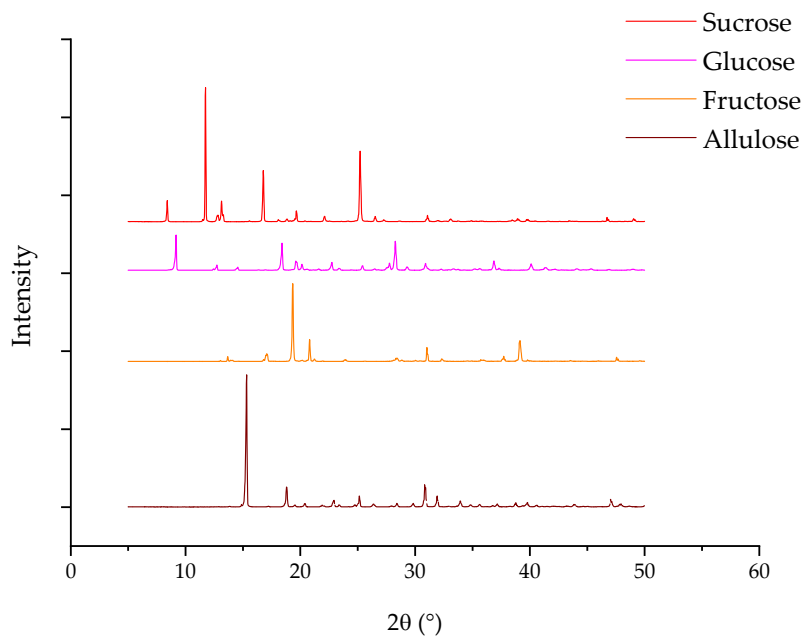

**Figure S1.** X-ray Diffraction (XRD) spectrum of sugars in solid form.

Supplement: Supplementary file 1 [file foods-11-01148-s001.zip › foods-1634432-supplementary.pdf]
